# Dementia and Imagination: A Social Return on Investment Analysis Framework for Art Activities for People Living With Dementia

**DOI:** 10.1093/geront/gny147

**Published:** 2018-11-23

**Authors:** Carys Jones, Gill Windle, Rhiannon Tudor Edwards

**Affiliations:** Centre for Health Economics and Medicines Evaluation, Bangor University, Bangor,UK; Dementia Services Development Centre, School of Health Sciences, Bangor University, Bangor,UK; Centre for Health Economics and Medicines Evaluation, Bangor University, Bangor,UK

**Keywords:** Visual arts, Creativity, arts, and related therapy, Economics

## Abstract

**Background and Objectives:**

Arts activities may benefit people living with dementia. Social return on investment (SROI) analysis, a form of cost-benefit analysis, has the potential to capture the value of arts interventions, but few rigorous SROI analyses exist. This article presents a framework for an SROI analysis.

**Research Design and Methods:**

One hundred twenty-five people with mild to severe dementia and 146 caregivers were recruited to the Dementia and Imagination study across residential care homes, a hospital and community venues in England and Wales for a 12-week visual arts program. Quantitative and qualitative data on quality of life, support, and program perceptions were obtained through interviews. SROI was undertaken to explore the wider social value of the arts activities.

**Results:**

An input of £189,498 ($279,320/€257,338) to deliver the groups created a social value of £980,717 ($1,445,577/€1,331,814). This equates to a base case scenario of £/$/€5.18 of social value generated for every £/$/€1 invested. Sensitivity analysis produced a range from £/$/€3.20 to £/$/€6.62 per £/$/€1, depending on assumptions about benefit materialization; financial value of participants’ time; and length of sustained benefit.

**Discussion:**

To our knowledge, this is the first study applying SROI to an arts intervention for people with dementia. Arts-based activities appear to provide a positive SROI under a range of assumptions.

**Implications:**

Decision makers are increasingly seeking wider forms of economic evidence surrounding the costs and benefits of activities. This analysis is useful for service providers at all levels, from local government to care homes.

There are 47 million people living with dementia worldwide, and this is predicted to rise to more than 131 million by 2050 (Prince, Comas-Herrera, Knapp, Guerchet, & Karagiannidou, 2016). In the United Kingdom, two-thirds of the cost of dementia is paid by people with dementia and their families, and unpaid caregivers supporting someone with dementia save the economy £11 billion ($16.2/€14.9 billion) a year (Knapp et al., 2014).

There is an emerging body of evidence that arts-based activities can be enjoyable and have other benefits for people living with dementia. Research reviews suggest that art interventions have the potential to improve a broad range of outcomes for people living with dementia, including well-being, quality of life, cognitive function, and communication (De Medeiros & Basting, 2014;Mental Health Foundation, 2011;Young, Camic, & Tischler, 2016;Young, Tischler, Hulbert, & Camic, 2015;Zeilig, Killick, & Fox, 2014). Such programs could be developed as part of dementia services at a local level if they are proven to yield direct benefits to people with dementia and have wider societal benefits.

The National Institute for Health and Care Excellence (NICE) in the United Kingdom has typically supported cost-utility analysis using quality-adjusted life years (QALYs) as the metric of benefit for clinical studies. QALYs are a composite measure of health-related quality of life, which combine the length of life gained as the result of an intervention with the quality of life associated with being in a particular health state. As increasing length of life is not always the aim of psychosocial interventions, QALYs may not be the most relevant metric to use. The use of cost-benefit, cost-consequence, and return on investment analysis have also been deemed appropriate for capturing outcomes of interventions that include nonhealth benefits, such as benefits to caregivers (NICE, 2011,^2012^). This approach is particularly relevant for interventions that support people living with dementia, where benefits may appear to be “in the moment” and are hard to capture using a cost-utility framework. Benefits to caregivers are clearly relevant given the significant contribution of informal care to the provision and total cost of dementia (Knapp et al., 2014).

Where benefits fall across sectors, such as the health care sector, social care sector and local government, cost-benefit analysis, which measures all costs and benefits in monetary terms, has been advocated (McIntosh, Donaldson, & Ryan, 1999). Social return on investment (SROI) analysis is a pragmatic form of cost-benefit analysis which seeks to establish the social value generated by an intervention (Inglis, 2012;Nicholls, Lawlor, Neitzert, & Goodspeed, 2009;The Centre for Public Scrutiny, 2014). SROI attempts to capture a broader picture of the value of an intervention by considering the impact on the locality of the intervention and incorporating social value where appropriate. A triple bottom line approach is taken, meaning the effect on the economy, the environment and its people are considered.

SROI analysis has been used to assess the social value of creative activities for older people (MB Associates, 2013;Social Value Lab, 2011). These activities were found to generate a positive return on investment, meaning that the social value generated was greater than the value invested in setting up and delivering the activities. However, the social value of arts groups for people living with dementia has not been established. SROI does not yet have the pedigree of cost-benefit analysis (Fujiwara, 2015) and the method, it may be argued, is still seeking academic credibility in terms of a need to build (a) a standardized methodology and (b) a body of robust published examples of its application. However, the method has been advocated by the U.K. cabinet office (Nicholls et al., 2009), and there is some interesting comparable work in the Netherlands on the social cost-benefit analysis of public projects with a potential to improve population health (de Wit et al., 2016).

## The Present Study

“Dementia and Imagination” was a mixed-methods longitudinal study, exploring the impact of a visual arts program on people living with dementia. The analyses found that across all sites (hospital, community, residential care), scores for the well-being domains of interest, attention, pleasure, self-esteem, negative affect, and sadness were significantly better in the art program than the alternative social activity control condition. Proxy-reported quality of life (QoL) significantly improved between baseline and 3-month follow-up, but no improvements in QoL were reported by the participants with dementia. This was contrasted by their qualitative accounts, which described a stimulating experience important for social connectedness, well-being, and personal resilience. Communication deteriorated between baseline and follow-up in the hospital setting but improved in the residential care setting (Windle et al., 2016;Windle, Joling, et al., 2018). We present here the novel SROI analysis framework used to explore the economic impact and social value generated by the arts activities.

## Methods

Dementia and Imagination was a nonrandomized mixed methods longitudinal cohort study with all recruited participants offered the intervention. Participants were recruited between May 2014 and May 2015. Assessments were carried out by researchers at baseline, 12 weeks, and 6 months using a mixture of qualitative interviews and quantitative measures. The study protocol provides in-depth methodological details (Windle et al., 2016), and findings relating to the quality of life, communication, and well-being are available elsewhere (Windle, Joling, et al., 2018).

### Participants and Settings

Research site 1 was comprized of four residential care facilities in the North East of England. Site 2 was two assessment units within a National Health Service (NHS) county hospital in Derbyshire. Here, the protocol was modified after the second wave of intervention delivery to also include recruitment from a daycare service for people with dementia. Site 3 involved three community venues in North Wales (library with a small exhibition area, an arts center with a gallery, an international arts and music venue) with participants recruited to this site through primary and secondary care services. Before recruitment, the study calculated that a 95% confidence level with 5% margin of error and a moderate effect size would require*n* = 80. To adjust for attrition, the study sought to over-recruit by at least 25%, resulting in a total required number of 100 participants living with dementia and 100 caregivers (Windle et al., 2016;Windle, Joling et al., 2018).

Participants living with dementia were included if they had a diagnosis of dementia or evidence of age-related memory impairment and were:

- A resident in the chosen care home in Newcastle/Tyne and Wear.
- A resident in the assessment unit/in receipt of services for a minimum of 3 months in Derbyshire.
- Living in the community in rented/private housing or sheltered housing in North Wales.

Participants were excluded if they had a recent or current episode of major mental illness (other than dementia), were at the end of life or terminally ill, had a debilitating illness that would preclude regular attendance, had a severe uncorrected sensory or communication difficulty and were completely unable to communicate verbally through the medium of either English or Welsh.

Professional and family caregivers were recruited between May 2014 and May 2015 alongside the recruitment activity for people living with dementia. Participants were included if they were a member of staff in the residential care homes or National Health Service (NHS) facility who had regular contact with the participant living with dementia, or spouse, family member, or friend of the participant living with dementia (the primary caregiver). Exclusion criteria were recent or current episode of major mental illness, end of life/terminal illness, and inability to communicate verbally through the medium of either English or Welsh. The caregivers were involved in two ways. The first was to provide proxy data on behalf of the participant with dementia, should they be unable to do so themselves. The second was to provide data on the impact of the program on their own perceptions of the person living with dementia.

All participant information provided was prepared to be simple, clear, and understandable. Bilingual information (Welsh and English) was prepared in Wales. Researchers met with potential participants and family or professional caregivers to explain the study. Qualitative and quantitative data were collected concurrently through an interview at baseline before starting the 12-week program, and follow-up interviews were conducted 3 months (Time 2) and 6 months (Time 3) later.

### Ethical Approval

Ethical approval was granted by the Multi-centre Research Ethics Committee (MREC) for Wales (ref. 13/WA/0365) on February 14, 2014. Each site received approval from their Local Research Ethics Committee (LREC) and for the North Wales and Derbyshire settings, the appropriate NHS Trust Research and Development department.

### The Intervention

The intervention program was developed through a theoretical investigation of the contextual factors and mechanisms which shape outcomes (Windle, Gregory, et al., 2018) and builds on identified good practice, such as those offered by national galleries, for example, Museum of Modern Art, New York; National Gallery of Australia. This work was adapted into the working principles of the intervention and standardized as the guidelines for intervention delivery.

The program comprised two underpinning factors; dynamic and responsive artistic practice, and a provocative and stimulating aesthetic experience. These were implemented through the content of seven key ingredients for excellent practice: (a) artists understanding dementia; (b) developing a safe and supportive physical and psychological environment for an inspirational visual arts viewing and making program; (c) creating a structure for the viewing and making sessions; (d) delivering sessions that enable inspiration, imagination, creativity, enjoyment, and celebration; (e) developing social connections; (f) personal development; and (g) values, ethics, communication, and guiding principles.

The delivery of the program involves participative activities with the emphasis on providing a stimulating, high-quality experience for the participants, requiring no prior knowledge or skills. It aims to encourage creativity without overwhelming people with complex instructions, be interesting and challenging and promote learning where possible. It encompasses meaningful engagement to stimulate imagination, play, and discussion, not lectures or the generation of factual exchanges reliant on memory for names and dates. It provides some structure but creates the opportunity for individual expression, fun, and celebrations of achievements in a failure-free environment.

A lead artist with prior experience and training in art and dementia facilitated each session, supported by a second artist. Generally, the sessions were structured so that the first half was an art viewing activity, focusing on a small number of artworks, followed by art-making, however this was flexible and dependent upon the varying degrees of cognitive impairment presented to the artists. Different materials were provided depending on the art-making task, such as water-based paints, pastels, color pencils, collage material, glue, iPad, quick-drying modeling clay, and print-making supplies.

Up to 12 people attended each intervention group, and 11 groups were delivered in total across the three settings. One group consisted of 12 weekly sessions of 2 hr. Each care home in Site 1 had one visit to a local gallery. No gallery visits were made in Site 2 due to restrictions on staff leaving the hospital. Where gallery visits were not possible, the artists brought a small selection of artworks to the participants to facilitate discussions. In Site 3, the community libraries had small exhibition areas facilitating art viewing. In the art center, the collection was visited each week. Caregivers and staff were not required to take part in the intervention, although some chose to do so. A postintervention review meeting with the artists indicated the program was delivered according to the core principles, and a practitioners’ guide, co-produced with the artists, is freely available (Parkinson, Windle, & Taylor, 2017).

### Social Return on Investment

An evaluative SROI analysis was undertaken. SROI analysis involves several steps; establishing scope and involving stakeholders, mapping outcomes, evidencing and valuing outcomes, establishing impact, and calculating the SROI ratio. Each step is explained in detail below.

#### Establishing scope and involving stakeholders

Stakeholders are the people or organizations that are materially affected by an activity. For the purposes of our analysis, people living with dementia, their families, and staff caregivers are included as stakeholders (Table 1). The financial input of state/partner organizations was included; however, no material outcomes were assumed for this stakeholder group. In total, 125 people with dementia and 146 caregivers (88 family and 58 staff caregivers) were included in the analysis.

**Table 1.** Stakeholders Included and Not Included in this Analysis

Stakeholder	Included/excluded?	Reason for including/excluding	What the stakeholder invested
The state	Inputs included, outcomes excluded	The state acted as a funder for the art groups.	The running costs for the groups (excluding research costs) was £103,292 ($152,252/€140,271)
Partner organizations	Inputs included, outcomes excluded	Partner organizations may have experienced an increase in people seeking information and increased footfall at galleries, but this was not measured by the study.	Partner organizations contributed £44,846 ($66,103/€60,901) of in-kind contributions, e.g., use of venues, promoting and curating exhibitions
People living with dementia	Included	People attending the groups were the primary stakeholders of the intervention.	Time: 3 hr per session (1 hr travel/ organization, 2 hr group) @ minimum wage of £7.20 ($10.61/€9.62) per hour = £19,634 ($28,941/€26,663)
Family caregivers	Included	It was anticipated that there could be an impact on art engagement, social connectivity, and attitudes toward dementia for families following participation of a loved one in the arts groups.	Time: 2 hr of organizational tasks per session attended by a loved one @ £19 ($28/€26) per hour, the cost of a home care worker = £13,090 ($19,294/€17,776)
Staff caregivers	Included	It was anticipated that regular contact with participants attending the groups could indirectly lead to increased engagement with art and a change in attitude toward people with dementia in the staff working in the hospital and care home settings.	Time: 0.5 hr of organizational tasks per person with dementia in their care attending @ £19 ($28/€26) per hour, the cost of a home care worker = £8,636 ($12,729/ €11,728)
Artists delivering the sessions	Excluded	The artists had experience of working with people with dementia. While their involvement could have led to skill development and greater employment opportunities, it was anticipated that there would be a negligible material impact.	
NHS	Excluded	The program was developed with a goal of connecting communities and improving well- being. While it is possible that the benefits could extend to a reduction in participants’ health care use, the study did not collect this data. As such, it was not feasible to include the NHS as a stakeholder.	
Nonparticipating residents at care homes/ assessment units	Excluded	It was possible that the program resulted in a more positive environment within participating sites; however, as nonparticipants did not meet the inclusion criteria of the study or did not consent to take part it was unethical to include them.	
Participants’ wider social networks	Excluded	It was beyond the scope of the project to map participants’ wider social networks.	
General public residing in the three study settings	Excluded	Public engagement can be a first step in raising awareness but changes in attitudes may require more targeted interventions. A number of engagement/dissemination activities took place but their impact on the public are not included in this analysis.	

For transparency, we describe inTable 1 other groups and organizations that were considered as stakeholders, but subsequently excluded from the analysis. Boundaries needed to be established over what was feasible to measure and include, and exclusion was either on the grounds of there being no material impact expected on the group, or their involvement was outside of the scope of the evaluation.

#### Mapping outcomes

A theory of change was developed (Supplementary Figure 1), which represented how the arts activities were expected to bring about change for the key stakeholders. It was developed from the findings from the theoretical investigation (Windle, Gregory, et al., 2018) and consultation with artists and people with dementia who had previous experience of taking part in art groups. Working with stakeholders to identify the impacts of taking part in the activities under evaluation is a core component of SROI analysis.

#### Evidencing outcomes

A mixture of quantitative and qualitative data were collected as part of Dementia and Imagination; a full list of measures is available in the study protocol (Windle et al., 2016). For the purposes of the SROI analysis, a list of measures were identified that would capture changes in the outcomes identified during the mapping outcomes stage. These included standardized measures and individual items.Table 2 outlines the list of outcomes by stakeholder group, how changes in the outcomes were measured, and how changes were valued. These included single items (to reflect the topics identified in the initial work with stakeholders) derived from demographic data and the 28-item DEMQOL dementia-specific quality of life measure (Smith et al., 2005) with higher values indicating higher quality of life, and questions on their extent of engagement with art.

**Table 2.** Outputs, Outcomes, and Sources of Information for Indicators and Financial Proxies

Stakeholders	Intended/unintended changes	Outputs	The outcomes (what changes)						
Who will we have an effect on?		Summary of activity in numbers	Indicator: How we measured outcomes	Source	Quantity: How much change was there?	Duration: How long change lasts (years)	Financial Proxy	Value	Financial proxy source for valuing outcomes
The state/partner organizations	N/A	132 D&I sessions delivered	Records of number of sessions delivered	Records of number of sessions delivered	132	1	Average cost per group (£783/$1,154/€1,063) and average in-kind contribution per group (£340/ $501/ €462)	£1,123 ($1,713/€1,578)	Intervention costs, supplemented by information from weekly diaries completed by the artists describing time and materials used
People living with dementia	Increased well-being/ improved mood	(36/98) 36.7% experienced an increase in wellbeing	Change in DEMQoL total score between baseline and T3	Interviews with participants and proxies	36	1	HEA1603: Good overall health age 50+	£20,323 ($29,956/€27,599)	HACT social value bank
	Increased engagement with art	(53/100) 53% reported a maintain or increase in art activities	At baseline, art activities in last 12 months were recorded (visits to museums, galleries). At T3, participants were asked if they had taken part in art activities in the last few weeks		53	1	HOB1602: Hobbies age 50+	£2,424 ($3,573/€3,292)	HACT social value bank
	Increased confidence/ self-esteem	(17/61) 27.9% reported an increased confidence	Change in DEMQOL Q5 (“In the last week, have you felt confident?”) between baseline and T3		17	1	HEA1601: High confidence age 50+	£12,565 ($18,521/€17,063)	HACT social value bank
	Increased feeling of control over their life/personal environment	(18/61) 29.5% reported an increased their feeling of control	Change in DEMQOL Q13 (“In the last week, have you felt that there have things that you wanted to do but couldn’t?”) between baseline and T3		18	1	HEA1406: Feel in control of life age 50+	£16,427 ($24,213/€22,308)	HACT social value bank
	Reduced social isolation/increased sense of belonging	(6/58) 10.3% reported a decrease in loneliness	Change in responses to the question “Do you feel lonely?” between baseline and T3		6	1	ENV1609: Feel belonging to neighborhood age 50+	£6,004 ($8,850/€8,153)	HACT social value bank
	Increased physical activity	(21/98) 21.4% reported an increase in liveliness	Change in DEMQOL Q10 (“In the last week, have you felt lively?”) between baseline and T3		21	1	SPO1607: Frequent mild exercise age 50+	£5,527 ($8,147/€7,506)	HACT social value bank
Families/friend caregivers	Increased engagement with art	(27/54) 50% reported arts activities at T3	Response to questions about involvement with art outside of work/home at T3	Interviews with caregivers	27	1	HOB1602: Hobbies any age	£1,515 ($2,233/€2,057)	HACT social value bank
	Increased social support network	(13/47) 27.7% reported increased social network	Response to T3 question about whether the person has kept in touch with people involved with the art groups		13	1	ENV1609: Feel belonging to neighborhood any age	£3,753 ($5,532/€5,097)	HACT social value bank
	Change in attitude toward participants	(34/70) 48.6% had an improved attitude toward dementia	Changes in Approaches to Dementia Questionnaire total score between baseline and T3		34	1	EMP1610: General training for the job	£1,567 ($2,310/€2,128)	HACT social value bank
Care home staff	Increased engagement with art	(23/29) 79.3% reported arts activities at T3	Response to questions about involvement with art outside of work/home at T3	Interviews with caregivers	23	1	HOB1602: Hobbies any age	£1,515 ($2,233/€2,057)	HACT social value bank
	Opportunity for professional development/ increased feeling of prestige	(28/33) 84.8% reported a change in their thinking about working practices OR a positive perception of visitors to the home	Response at T3 to questions about whether they have identified ways of working that they can improve on, and whether there has been a change in the way visitors view their workplace		28	1	EMP1611: Employment training any age	£807 ($1,190/€1,096)	HACT social value bank
	Change in attitude toward participants	(18/45) 40% had an improved attitude toward dementia	Changes in ADQ total score between baseline and T3		18	1	EMP1610: General training for the job	£1,567 ($2,310/€2,128)	HACT social value bank
	Increased community engagement	(11/20) 55% reported increased social network	Response to T3 question about whether the person has kept in touch with people involved with the art groups		11	1	ENV1609: Feel belonging to neighborhood any age	£3,753 ($5,532/€5,097)	HACT social value bank

Family and staff caregivers completed the 31-item DEMQOL-proxy, demographic questions, a self-reported health item, the 19-item Approaches to Dementia questionnaire (Lintern & Woods, 1996), and questions on perception of art activity.

The proxy responses were used when the responses for the person living with dementia were not reported. Specifically, 37 proxy responses were used for the change in the DEMQoL total score between baseline and T3 and the question “in the past week have you felt lively,” and 44 proxy responses were used for the question on increased engagement with art.

#### Valuing outcomes

Table 2 presents the associated data for this step of the SROI analysis. Unit prices in U.K. sterling for the year 2015/2016 were applied to the data. Currency conversion rates for January 1, 2016 have been applied to present equivalent $/€ values (https://www.xe.com/currencytables/). Artists were asked to complete diaries to record the materials, time and in-kind contribution (e.g., use of venues for delivering the groups or exhibition space). The associated costs for the artists’ time were not included as this was part of the program costs paid for by the state stakeholder and including it again here would be double counting.

Participants’ time input was included in the analysis, with an assumption of 3 hr per session per person with dementia, representing 2 hr of activity, with an extra hour added to account for traveling to and from the sessions and/or getting prepared to attend. Two hours per session per family caregiver were assumed to account for organization and transport tasks, and 0.5 hr per staff caregiver to account for organization tasks (as the sessions were “on site” no travel time or dead time while people attended sessions was included). These assumptions were derived through initial scoping work by the lead author, who: (a) visited a similar arts group elsewhere in the country to discuss organization, and (b) met a person living with dementia and their caregiver who had taken part in a similar arts group.

The value of this time input was calculated as £7.20 ($10.61/€9.62) per hour for people with dementia, based on the U.K. National Minimum Wage in 2016, which reflects the assumption that most home care workers are on this minimum wage. Nineteen pounds ($28/€26) was assumed as the hourly cost of replacing/buying equivalent per hour for family and staff caregivers, the average cost of a home care worker (Curtis & Burns, 2015).

Financial proxies were then applied to the observed outcomes choosing proxies is very subjective, and is a challenge of SROI. Financial proxies can be obtained through including additional questions on participants’ willingness to pay, however, to minimize participant burden we instead used where possible a databank of social value proxies that have been derived using consistent methodology from sources including national surveys and the U.K. census.Table 2 presents the sources of the proxies. The primary source of financial proxies was the HACT Social Value Bank (http://www.hact.org.uk/social-value-bank). This is a databank of methodologically consistent unit costs for social value indicators. In economic evaluation, it is typical to apply a discount rate to adjust future costs and outcomes to present values. NICE recommends a discount rate of 1.5% for public health interventions (NICE, 2012) as these typically show effects over a long-term time horizon. As dementia is a progressive condition we considered the U.K. Treasury recommended discount rate of 3.5% for costs and outcomes occurring after 1 year to be more appropriate for this study.

## Results

The average age of the participants living with dementia was 81.4 (*SD* = 8.5). Fifty-eight percent (*n* = 73) were female, 64% (*n* = 71) had a low level of education, 45% (*n* = 56) were married and 43% (*n* = 52) were widowed. Across sites, the participants attended an average of seven sessions (*SD* = 3.83). The family caregivers were older (*M* = 63.3,*SD* = 14.53) than the professional caregivers (*M* = 46.5,*SD* = 13.93). Most of the caregivers were female (79%,*n* = 116), and 62% (*n* = 90) were married.

People living with dementia, their family caregivers and staff caregivers all experienced increased engagement with art, leading to a modest generation of social value (Supplementary Table 1). Half of the staff caregivers reported increased engagement with their local community and 85% reported professional development or improved prestige associated with their work as a result of improved visitor perceptions of the care home. The outcome that led to the most social value was improved well-being for people with dementia, which generated a social value of £373,350 ($550,318/€507,009). This was followed by increased feeling of control over their life/environment, which generated the social value of £150,889 ($222,410/€204,907); and increased confidence, which generated the social value of £109,003 ($160,670/€148,026).

The inputs and outcomes for each stakeholder group (Table 2) were transferred on to an impact map adapted fromNicholls and colleagues (2009), which indicated the scale of material changes for each stakeholder group, and the associated value that was generated (Supplementary Table 1).

### Establishing Impact

In SROI analysis, the choice of financial proxies, and indeed the stakeholders and outcomes to include, is subjective. To minimize the risk of over claiming benefits, deadweight, displacement, attribution, and attrition are included in the analysis. Deadweight is the proportion of change that people would experience over time, regardless of taking part in the study. For people living with dementia, this could be a decline in quality of life that would be expected over time. We make this assumption based on observations that health-related quality of life in the United Kingdom declines for the population as a whole as measured by the EURQoL group (https://euroqol.org/).

Displacement is the proportion of change that is being displaced, for example, the care homes canceling or rearranging other activities to make way for the arts groups. Attribution is the proportion of the observed change that is due to taking part in the arts groups, rather than being the outcome from another hobby or activity that the participants were doing. In SROI, attrition refers to the proportion of effects that drop-off after the first year, rather than being the attrition rate of people taking part in the study. Deadweight, displacement, attribution, and attrition were measured through questions asked to the participants about their level of activity at baseline, after completing the 12-week art group, and 3 months later (Supplementary Table 1).

A series of sensitivity analyses were conducted to address the subjective nature of the financial proxies, which include an assumption of £0 cost for participants’ and caregivers’ time, only 50% of observed outcomes materializing, outcomes lasting for 2 years instead of 1 year, and assuming that the financial proxy used for a year of well-being was 75% lower (Table 3).

**Table 3.** SROI Analysis Results

Scenario	SROI ratio (£/$/€)
Base case	5.18: 1
Assuming a £0 value for the time of people with dementia, their families and staff caregivers	6.62: 1
Assuming only 50% of outcomes materialize for people with dementia	3.20: 1
Assuming only 50% of outcomes materialize for family caregivers	4.95: 1
Assuming only 50% of outcomes materialize for staff caregivers	5.01: 1
Assuming all outcomes last up to 2 years instead of 1 year	6.36: 1
Assuming the financial proxy for a year of well-being is 75% lower	3.75: 1

### Calculating the SROI Ratio

The value of inputs over the 132 art group sessions was £189,498 ($279,320/€257,338) and the value of outputs was £980,717 ($1,445,577/€1,331,814), leading to a base case scenario of £/$/€5.18 of social value generated for every £/$/€1 invested in Dementia and Imagination.

To test the robustness of the results, a range of scenarios are presented inTable 3. When we tested a scenario that only 50% of observed outcomes materialized for people with dementia, the SROI ratio resulted in £/$/€3.20 of social value for every £/$/€1 invested. Assuming that only 50% of outcomes materialized for family and staff caregivers had less of an impact, with the SROI ratio changing to 4.95:1 and 5.01:1, respectively.

Our base case analysis took a cautious approach and assumed that outcomes lasted for 1 year as the health and well-being of participants with dementia is likely to decrease over time. Changing this assumption to outcomes lasting for 2 years resulted in a higher SROI ratio of 6.36:1. All tested scenarios resulted in a positive SROI ratio, meaning that for every £/$/€1 invested in the arts activities over £/$/€1 of social value was generated in return.

## Discussion

To our knowledge, this is the first study applying SROI analysis to an arts-based intervention for people living with dementia. Arts-based activities appear to provide a positive and convincing SROI under a range of assumptions.

We found a social value of between £/$/€3.20 and £/$/€6.62 for every £/$/€1 invested in the arts groups, with a base case scenario of 5.18:1. The highest proportion of this social value was generated for the stakeholder group of people living with dementia. The sensitivity analysis scenario of assuming that only 50% of their observed outcomes materialized resulted in the lowest SROI ratio (3.20:1). The highest SROI ratio was found in the scenario where the value of time for all stakeholder groups was assumed to be £/$/€0 (6.62:1); however, we believe this to be a scenario (e.g., a volunteer scenario) where the costs are underestimated due to the economic concept of opportunity cost. Opportunity cost means that when investing time, money, or other resources into a particular activity one has to forego investing those resources in another activity. In this evaluation, we assigned a value to participation time because if our stakeholders had not have taken part in the study they could have spent their time on leisure activities, volunteering or on other tasks.

In terms of comparing our findings with previous related studies, two SROI analyses of craft activities for older people found ratios of 3:1 for a program of training care home staff in creative activities (MB Associates, 2013), and 8.87:1 for a community craft café in Scotland (Social Value Lab, 2011). Increased independence for people taking part in the arts activities and workforce development for staff caregivers were identified as stakeholder outcomes; the Dementia and Imagination study found similar outcomes. The positive findings from our SROI analysis also support findings from the main effectiveness analysis of the study; across all sites, scores for the wellbeing domains of interest, attention, pleasure, self-esteem, negative affect, and sadness were significantly better in the arts intervention compared to an alternative activity with no art (Windle, Joling, et al., 2018).

For people living with advancing dementia who may be unable to provide answers to standardized questionnaires, obtaining their thoughts and preferences from a family member or professional caregiver through a proxy assessment (as with some of our data collection) is a common approach for the evaluation of people with dementia. There is some suggestion that this may at times be prone to some bias, with caregivers under-reporting quality of life compared to the reports from people living with dementia (e.g.,Crespo, Bernaldo de Quirós, Gómez, & Hornillos, 2012). However, this was not found in Dementia and Imagination; caregivers reported significant improvements over time in their proxy assessments of quality of life, whereas there was no change over time in quality of life as reported by the people living with dementia (Windle, Joling et al., 2018).

The Dementia and Imagination study and the two previous studies we mention above should be viewed in the context of an aging population, of whom 16% have reported experiencing loneliness (O’Luanaigh et al., 2008). The effects of loneliness and depression are particularly significant. There is a strong association between loneliness and depression with an associated detrimental effect to physical health, affecting blood pressure levels, sleep, the immune system, and cognition; the strength of social networks, including arts activities, could be of particular significance in this respect.

While the cost-per-QALY metric is considered the gold standard for economic evaluations of health technologies (Hughes et al., 2016;NICE, 2013), NICE endorses the use of cost-benefit analysis to capture a wider range of health and nonhealth impacts in public health interventions (NICE, 2012). We feel it useful to think of SROI analysis as a pragmatic form of cost-benefit analysis, appropriate for assessing the social value of initiatives, while taking into account the distinctiveness of specific contexts. However, this is also one of the limitations of both cost-benefit and SROI analyses; being specific to particular contexts restricts the generalizability of results and the ability to make direct comparisons between programs. Consequently, presenting the data used to calculate the ratio in a transparent manner is vital for allowing readers to interpret the rigor and validity of any SROI study.

While the number of studies using SROI analysis is growing, few SROI analyses are currently published in peer-reviewed journals and findings are typically presented as gray literature (reports published on the funders’ websites or as commentaries on policy). Consequently, this approach is yet to be fully utilized and established internationally. The quality of available evidence is variable (Banke-Thomas, Madaj, Charles, & van den Broek, 2015) and the methodology used has been open to criticism (Fujiwara, 2015), spanning: (a) a lack of a normative basis; (b) challenges of making interpersonal comparisons about benefit accrual; (c) the choice of the number and range of stakeholders may be viewed as subjective; (d) the need for transparency to explain how SROI ratios are calculated to avoid concerns over bias; (e) statistical methods for inferring causality are problematic in SROI; (f) a need for continued work on valuation methods; (g) challenges comparing across projects, due to the lack of standardization in SROI methods; and (h) the subjective nature of the selection of outcomes and financial proxies.

These criticisms can be summed up as we have argued in our introduction, in the need for (a) an increasingly standardized SROI methodology and (b) a body of robust published applications of SROI—our article contributes to this latter goal. While we recognize the limitations of the approach, we have presented the steps outlining how the stakeholders for this analysis were selected, the underlying theory of how the arts activities bring about change (Supplementary Figure 1), comprehensive information on how material changes were identified using quantitative and qualitative measures, and the sources of financial proxies used to value outcomes. In doing so, we hope this article contributes new insights into this developing area of economic analysis.

### Implications

Decision makers are increasingly seeking wider forms of economic evidence surrounding the costs and benefits of activities. This SROI analysis of the Dementia and Imagination art program for people with dementia is useful for service providers at all levels, from local governments delivering arts programs, to individual care homes looking at how best to invest their activities budget. The detailed analysis allows readers to interpret which elements of the activities generated the most social impact, which has relevance for service providers worldwide.

Given that the annual global economic impact of dementia on society is estimated at US$604 billion (World Health Organisation, 2017), services worldwide are faced with incredible challenges regarding the prioritization of limited budgets to services. This novel evaluation, underpinned by a theoretical model explaining how arts programs may benefit people with dementia, demonstrated the positive benefits of arts activities for people with dementia, their families, and staff working with people with dementia, and as such provides useful information for those planning dementia services.

## Funding

Dementia and Imagination was funded as “Dementia and imagination: connecting communities and developing well-being through socially engaged visual arts practice,” Grant Ref: AH/K00333X/1, by the AHRC and ESRC as a part of the Cross-Council Connected Communities Programme.

## Supplementary Material

GERONTOLOGIST_60_1_112_s1
