# Supplementary material for: Dementia and Imagination: A Social Return on Investment Analysis Framework for Art Activities for People Living With Dementia
Source: Gerontologist. 2018 Nov 23;60(1):112–23. doi: 10.1093/geront/gny147 (PMC12774629; doi:10.1093/geront/gny147)
Supplement: GERONTOLOGIST_60_1_112_s1 [file gerontologist_60_1_112_s1.docx]

Supplementary Figure 1: Conceptual model of how visual arts programs may work, adapted from Windle et al. 2017


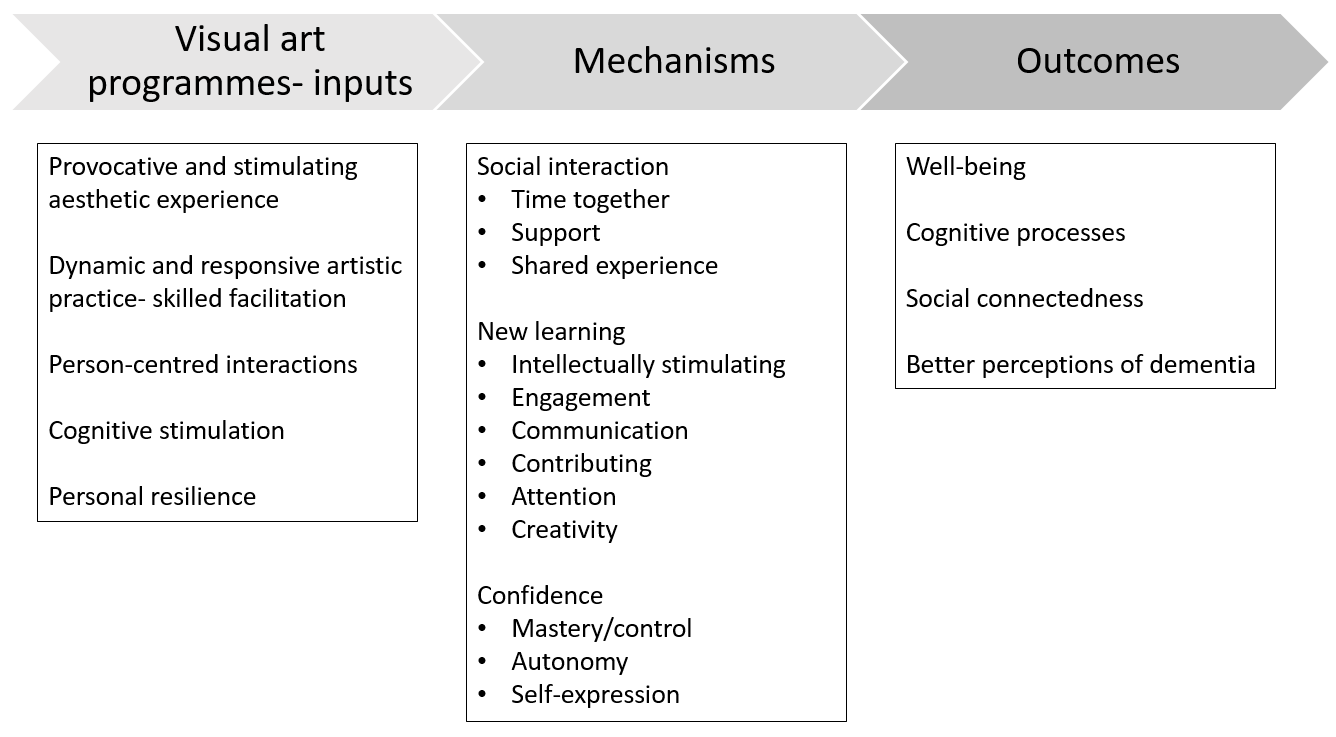


Supplementary Table 1: Impact map calculations for the analysis

| **Stakeholders** | **Input** | **Outcome** | | **Deadweight %** | **Displacement %** | **Attribution %** | **Drop off %** | **Impact** |
| --- | --- | --- | --- | --- | --- | --- | --- | --- |
|  |  |  | Value | What would have happened without the program? | What activity was displaced? | Who else contributed to the change? | Drop off in future years? | Quantity times financial proxy, less deadweight, displacement and attribution |
| The state/ partner organizations | £103,292 ($152,252/ €140,271) | 132 sessions delivered | £783 ($1,154/ €1,063) | 18% | 20% | 15% | 80% | £57,596 ($84,897/ €78,215) |
|  | £44,846 ($66,103/ €60,901) |  | £340 ($501/ €462) | 18% | 20% | 15% | 80% | £25,006 ($36,859/ €33,958) |
| People with dementia | £19,634 ($28,941/ €26,663) | Increased well-being/ improved mood | £20,323 ($29,956/ €27,599) | 10% | 30% | 19% | 80% | £373,350 ($550,318/ €507,009) |
|  |  | Increased engagement with art | £2,424 ($3,573/ €3,292) | 10% | 30% | 19% | 80% | £65,559 ($96,634/ €89,029) |
|  |  | Increased confidence/ self-esteem | £12,565 ($18,521/ €17,063) | 10% | 30% | 19% | 80% | £109,003 ($160,670/ €148,026) |
|  |  | Increased feeling of control over their life/ personal environment | £16,427 ($24,213/ €22,308) | 10% | 30% | 19% | 80% | £150,889 ($222,410/ €204,907) |
|  |  | Reduced social isolation/ increased sense of belonging | £6,004 ($8,850/ €8,153) | 10% | 30% | 19% | 80% | £18,383 ($27,097/ €24,964) |
|  |  | Increased physical activity | £5,527 ($8,147/ €7,506) | 10% | 30% | 19% | 80% | £59,229 ($87,304/ €80,433) |
| Families/friend caregivers | £13,090 ($19,294/ €17,776) | Increased engagement with art | £1,515 ($2,233/ €2,057) | 5% | 30% | 5% | 70% | £25,842 ($38,091/ €35,093) |
|  |  | Increased social support network | £3,753 ($5,532/ €5,097) | 5% | 30% | 5% | 70% | £30,822 ($45,432/ €41,856) |
|  |  | Change in attitude towards participants | £1,567 ($2,310/ €2,128) | 5% | 30% | 5% | 70% | £33,658 ($49,612/ €45,708) |
| Care home staff | £8,636 ($12,729/ €11,728) | Increased engagement with art | £1,515 ($2,233/ €2,057) | 13% | 30% | 15% | 20% | £18,038 ($26,558/ €24,496) |
|  |  | Opportunity for professional development/ increased feeling of prestige | £807 ($1,190/ €1,096) | 13% | 30% | 15% | 20% | £11,697 ($17,241/ €15,885) |
|  |  | Increased community engagement | £3,753 ($5,532/ €5,097) | 13% | 30% | 15% | 20% | £21,370 ($31,499/ €29,020) |
|  |  | Change in attitude towards participants | £1,567 ($2,310/ €2,128) | 13% | 30% | 15% | 20% | £14,601 ($21,522/ €19,828) |
| Total | £189,498 ($279,320/ €257,338 |  |  |  |  |  |  | £1,015,042 ($1,496,172/ €1,378,427) |
|  |  |  |  |  |  |  |  |  |
|  |  |  |  | Present value of each year (after discounting at 3.5%) | | | | £980,717 ($1,445,577/ €1,331,814) |
|  |  |  |  | Net Present Value (PV minus the investment) | | | | £791,220 ($1,166,258/ €1,074,477) |
|  |  |  |  | Social Return on Investment | | | | 5.18:1 |
